# A palliative care approach for adult non-cancer patients with life-limiting illnesses is cost-saving or cost-neutral: a systematic review of RCTs

**DOI:** 10.1186/s12904-024-01516-1

**Published:** 2024-08-05

**Authors:** Katharina Janke, Yakubu Salifu, Siva Gavini, Nancy Preston, Amy Gadoud

**Affiliations:** 1 Division of Health Research, Centre for Health Inequalities Research, Faculty of Health and Medicine, Lancaster University, Lancaster, LA1 4AT UK; 2 Division of Health Research, International Observatory on End-of-life Care, Faculty of Health and Medicine, Lancaster University, Lancaster, LA1 4AT UK; 3 Lancaster Medical School, Faculty of Health and Medicine, Lancaster University, Lancaster, LA1 4AT UK; 4 Department of Surgical Gastroenterology, Sri Venkateswara Institute of Medical Sciences, Alipri Road, Tirupati, 517501 India

**Keywords:** Palliative care, End-of-life care, Health care costs, Health care expenditures, Life-limiting illness, Systematic review, Narrative synthesis

## Abstract

**Background:**

Patients living with life-limiting illnesses other than cancer constitute the majority of patients in need of palliative care globally, yet most previous systematic reviews of the cost impact of palliative care have not exclusively focused on this population. Reviews that tangentially looked at non-cancer patients found inconclusive evidence. Randomised controlled trials (RCTs) are the gold standard for treatment efficacy, while total health care costs offer a comprehensive measure of resource use. In the sole review of RCTs for non-cancer patients, palliative care reduced hospitalisations and emergency department visits but its effect on total health care costs was not assessed. The aim of this study is to review RCTs to determine the difference in costs between a palliative care approach and usual care in adult non-cancer patients with a life-limiting illness.

**Methods:**

A systematic review using a narrative synthesis approach. The protocol was registered with PROSPERO prospectively (no. CRD42020191082). Eight databases were searched: Medline, CINAHL, EconLit, EMBASE, TRIP database, NHS Evidence, Cochrane Library, and Web of Science from inception to January 2023. Inclusion criteria were: English or German; randomised controlled trials (RCTs); adult non-cancer patients (> 18 years); palliative care provision; a comparator group of standard or usual care. Quality of studies was assessed using Drummond’s checklist for assessing economic evaluations.

**Results:**

Seven RCTs were included and examined the following diseases: neurological (3), heart failure (2), AIDS (1) and mixed (1). The majority (6/7) were home-based interventions. All studies were either cost-saving (3/7) or cost-neutral (4/7); and four had improved outcomes for patients or carers and three no change in outcomes.

**Conclusions:**

In a non-cancer population, this is the first systematic review of RCTs that has demonstrated a palliative care approach is cost-saving or at least cost-neutral. Cost savings are achieved without worsening outcomes for patients and carers. These findings lend support to calls to increase palliative care provision globally.

## Background

Even though the provision of palliative care is considered an essential component of a modern healthcare system, globally only a minority of people in need of palliative care currently receive it [1]. Considering the substantial increase in health care expenditure at the end of life [2, 3], improving access to palliative care might be one of several instruments in a policy mix aimed at tackling rising health care costs.

Much of the evidence regarding the cost impact of palliative care is based on studies that include a majority of patients with cancer even though patients living with chronic diseases other than cancer such as cardiovascular disease or chronic respiratory diseases constitute the majority of patients in need of palliative care [1]. Two Cochrane reviews that examined a mix of patients with both cancer and non-cancer diagnoses concluded that the evidence is inconclusive [4, 5]. Non-Cochrane reviews of studies examining a mix of patients with both cancer and non-cancer diagnoses that include only randomised controlled trials (RCTs) also find that there is minimal or inconclusive evidence that a palliative care approach is cost saving [6, 7]. Reviews that include retrospective studies, on the other hand, tend to find that a palliative care approach reduces health care costs [8, 9]. The only review of RCTs that has focused on patients with non-cancer diagnoses alone found that palliative care reduced hospitalization and emergency department use but did not examine the impact of palliative care on total health care costs [10].

The dearth of reviews that focus on non-cancer patients and examine total health care costs means it is unclear whether palliative care can achieve cost savings for patients with a non-cancer diagnosis. Total health care costs capture resource use across all parts of the health care sector such as hospital care, primary care and community care. By expressing resource use in monetary terms they can provide a more comprehensive measure of resource use compared to non-monetary measures such as hospitalization, outpatient visit, GP visit or readmission. For example, a palliative care approach might reduce hospital use but not result in overall cost savings if it simply shifts care from hospitals to community settings.

Retrospective studies, included in some of the previous reviews, have a higher risk of bias. They are also less helpful in decision making as they investigate the effect of a palliative care approach in patients before their death whereas prospective studies examine individuals who are dying. Analysing data for patients before their death to inform decision making about patients who are dying can lead to biased results because the study populations and the time periods that are examined differ between studies of decedents and studies of the dying [11]. Reviews including lower quality studies reporting a balance of evidence towards cost-saving can be difficult to interpret.

The aim of this systematic review of RCTs is to determine the difference in costs between a palliative care approach and usual care in adult non-cancer patients with a life-limiting illness.

## Methods

The study was conducted using the narrative synthesis approach by Popay et al. [12]. The narrative synthesis allowed the textual, tabular, and graphical synthesis of the included studies about health care costs in adult non-cancer patients. It was then reported using The Preferred Reporting Items for Systematic Reviews and Meta-Analyses (PRISMA) 2020 guidelines [13]. The protocol was registered in PROSPERO (CRD42020191082).

### Eligibility criteria

#### Inclusion criteria were

1. RCTs: published full articles and abstracts with sufficient information on health care costs.
2. adult patients (≥ 18 years) with non-cancer life-limiting illnesses from the following non-cancer disease groups used in the Global Atlas of Palliative Care: lung diseases, heart diseases, cerebrovascular diseases, central nervous system diseases, diseases of liver, renal failure, HIV and dementia [1]. Studies including cancer and different age groups were included only when the data of adults with non-cancer life-limiting illnesses could be extracted separately.
3. A palliative care approach as defined by the World Health Organisation (2014) “an approach that improves the quality of life of patients and their families facing the problem associated with a life-threatening illness, through the prevention and relief of suffering by eans of early identification and impeccable assessment and treatment of pain and other problems, physical, psychosocial and spiritual” [14].
4. a comparator group of standard or usual care.
5. The main outcomes were health care costs such as hospital costs, hospice costs, costs borne by patients, or costs borne by patients’ family members and total costs, i.e. the sum of all health care costs included in a study.

#### Exclusion criteria were

1. articles not written in English or German.
2. retrospective studies, protocols, opinion pieces, editorials, or individual case reports.
3. economic modelling studies that did not report health care use.

### Information sources and search strategy

To ensure a comprehensive search, studies were identified from the following electronic databases: Medline, CINAHL, EconLit, EMBASE, TRIP database, NHS Evidence (including NHS Economic Evaluation Database), Cochrane Library, and Web of Science. Databases were searched from inception to 26th January 2023. Search terms were developed together with the academic librarian and tailored to each electronic database. Search terms for all databases are presented in Table 1. We also searched conference proceedings (2015 to 2021) of the European Association of Palliative Care (EAPC). Additionally, we checked the reference lists of all studies that satisfied inclusion criteria 2 to 5, i.e., the reference list checking included studies that were not RCTs such as other types of prospective studies as well as retrospective studies if they satisfied inclusion criteria 2 to 5.

**Table 1:** Search strategies

Database	Palliative care search terms	health care use search terms	Filters
Medline	MH “Palliative Care”; MH “Hospice and Palliative Nursing”; MH “Palliative Medicine”; MH “Terminal Care”; MH “Terminally Ill”; TI palliative; TI “end-of-life”; TI terminal*; TI hospice	MH “Health Care Costs”; MH “Health Expenditures”; MH “Costs and Cost Analysis”; MH “Cost–Benefit Analysis”; TI cost*; TI finance*; TI economic*; AB cost*, AB financ*; AB economic*	SubjectAge = All Adult: 19 + years Language = English Language = German
CINAHL	MH “Palliative Care”; MH “Hospice and Palliative Nursing”; MH “Terminal Care”; MH “Terminally Ill Patients”; TI palliative; TI “end-of-life”; TI terminal*; TI hospice care; AB palliative; AB “end-of-life”; AB terminal*; AB hospice care	MH “Costs and Cost Analysis; MH “Cost–Benefit Analysis”; MH “Health Care Costs”; MH “Health Facility Costs”; MW economics; TI cost*; TI finance*; TI economic*; AB cost*, AB financ*; AB economic*	SubjectAge = All adult Language = English Language = German
EconLit	TX palliative care; TX terminal care; TX “end-of-life”; TX hospice*; TX terminally ill	TX cost*; TX price*; TX spend*; TX expend*; TX financ*; TX economic*	Language = English Language = German
Embase	palliative therapy; palliative nursing; terminally ill patient; hospice care; palliative.ab; “end-of-life”.ab; terminal*.ab; hospice.ab; palliative.ti; “end-of-life”.ti; terminal*.ti; hospice.ti	“health care costs”; health expenditures.mp; exp “cost benefit analysis”; cost*.ab; finance*.ab; economic*.ab; cost*.ti; financ*.ti; economic*.ti	Adult 18 to 64 years Aged 65 + years English German
Trip	palliative care; hospice care; palliative medicine; terminal care; terminally ill	health care costs; health expenditures; financ*; economic; cost*	Primary research
NHS Evidence	“Palliative Care”; “Hospice Care”; “Palliative Medicine”; “Terminal Care”; “Terminally Ill”	“Health Care Costs”; “Health Expenditures”; “Costs and Cost Analysis”; “Cost Benefit”; Cost”; “Economic”	Secondary Evidence = > Economic Evavluations; Primary Research = > Economic Evaluations
Cochrane Library	MeSH “Palliative Care”; MeSH “Hospice and Palliative Care Nursing”; MeSH “Palliative Medicine”; palliative.ti; “end-of-life”.ti; terminal*.ti; hospice.ti; palliative.ab; “end-of-life”.ab; terminal*.ab; hospice.ab; palliative.kw; “end-of-life”.kw; terminal*.kw; hospice.kw;	MeSH “Health Care Costs”; cost*.ti; financ*.ti; economic*.ti; cost*.ab; financ*.ab; economic*.ab; cost*.kw; financ*.kw; economic*.kw	
Web of Science	TOPIC “Palliative Care; TOPIC “Hospice Care”; TOPIC “Palliative Medicine”; TOPIC “Terminal Care; TOPIC “Terminally Ill”; TITLE “Palliative Care”; TITLE “Hospice Care”; TITLE “Palliative Medicine”; TITLE “Terminal Care”; TITLE “Terminally Ill”	TOPIC “Health Care Costs”; TOPIC “Health Expenditures”; TOPIC “Costs and Cost Analysis”; TOPIC “Cost–Benefit”; TOPIC “Cost”; TOPIC “Economic”; TITLE “Health Care Costs”; TITLE “Health Expenditures”; Title “Costs and Cost Analysis”; TITLE “Cost–Benefit”; TITLE “Cost”; TITLE “Economic”	
EAPC	A manual search of the 2015 to 2021 conference proceedings of the European Association for Palliative Care		

All palliative care search terms were combined using the Boolean operator OR and all health care use search terms were combined using the Boolean operator OR. The results from these two searches were then combined using the Boolean operator AND

### Selection process

Results of the initial literature search were uploaded to Covidence, an online tool to support literature screening. After the removal of duplicates, two authors independently screened the titles and abstracts for relevance. Full texts of all potentially relevant studies were assessed independently by two authors. Disagreements were resolved by consensus or by consulting a third reviewer. For the reference list checking one reviewer screened the titles in the reference lists and then screened the abstracts of all relevant references identified from the title screening. Full texts of all potentially relevant studies identified from the reference list checking where then assessed collaboratively by two reviewers.

### Data collection process

Data from each study were entered on a data extraction form. Key fields on this form were study design, sample size, the definition of the patient population, type of palliative care approach, difference in the quantity of health care use between palliative care approach and usual care and methods for determining differences. All studies were extracted by one author with a second author independently cross-checking data extraction on 36% of the studies included and disagreements were resolved by discussion including with a third reviewer if necessary.

### Data items and effect measures

The main outcomes were health care costs such as hospital costs, costs borne by patients, or costs borne by patients’ family members. For each health care costs measure, the difference between a palliative care approach and usual care was extracted. If available, uncertainty measures were extracted, with confidence intervals presented in preference to p-values. Where a study did not report the difference but reported means for both patient groups, the difference was calculated if there was sufficient data.

### Quality assessment

The quality of the included studies was assessed using the Drummond checklist for assessing economic evaluations [15]. We omitted Question 7 (Where costs and consequences adjusted for differential timing?) as discounting was not relevant with time horizons of at most one year and Question 8 (Was an incremental analysis of costs and consequences of alternatives performed?) as our focus was on costs. Studies were classified as high, medium, or low quality. The quality assessment was carried out by a single reviewer. Two reviewers assessed 36% of included studies and disagreements regarding grading were resolved by discussion. No studies were excluded based on quality assessment.

### Synthesis methods

As there were differences in study population, care settings and measurement periods, we used a narrative synthesis approach following the guidance by Popay et al. [12]. The guidance describes the four main elements of a narrative synthesis: developing a theory of change; preliminary synthesis; exploring relationships within and between studies; and assessing the robustness of the synthesis. We addressed these four elements in an iterative process.

To develop a theory of change we consulted other reviews as well as primary studies. We found two potential mechanisms through which a palliative care approach might generate cost savings: 1) palliative care may reduce futile treatments by managing patients’ symptoms and in parallel improving patients’ understanding of their disease and establishing goals of care through optimal communication [5, 16, 17] or 2) home-based palliative care may replace hospital care, with the costs of home-based palliative care being more than offset by the reductions in hospital costs [4, 18]. The review mentioned in the background section that focused on patients with non-cancer diagnoses provides empirical support for the first step of mechanism 2): palliative care reduces hospitalization and emergency department use [10]. In an early iteration our review question included both healthcare utilisation and costs. As a result of identifying mechanism 2) which clarifies that utilisation is only an intermediate step, we refined our review question to focus on costs.

For the preliminary synthesis we used tabulation and vote counting. In a spreadsheet we completed one row for each relevant outcome with study characteristics such as study design, country, data collection period, diagnosis, type of palliative care approach, setting, sample size and measurement period and results such as our quality assessment, the outcome measure with and without palliative care, the difference between these and a categorisation of this difference as showing a palliative care approach to be cost-saving, cost-increasing or cost-neutral. We then counted how many rows fell into each of these categories both overall and within different subgroups delineated by the study characteristics.

To explore relationships within and between studies we examined moderator variables such as diagnosis, measurement period and type of palliative care approach. In a later iteration we adopted the cost-effectiveness plane, a standard tool to present the results of cost-effectiveness studies, to graphically present the relationships between costs and patient outcomes within studies and the relationships between studies.

To assess the robustness of the synthesis we reflected critically on the synthesis process. This reflection addressed the two key aspects of robustness: the methodological quality of the included studies and the methodology of the synthesis. In an early iteration our review included any type of prospective study. As a result of the critical reflection we changed our inclusion criteria to only include RCTs. As the literature contained several RCTs, lower quality prospective studies could be excluded from the review following the principle of best evidence introduced by Slavin [19]. Decisions made over the course of the synthesis process can be subjective. To minimise subjectivity, the synthesis was conducted by one reviewer and at the end of each iteration checked for consistency by the other reviewers. All reviewers then contributed to the decisions made for the next iteration of the synthesis process.

## Results

### Study selection

**Fig. 1:**
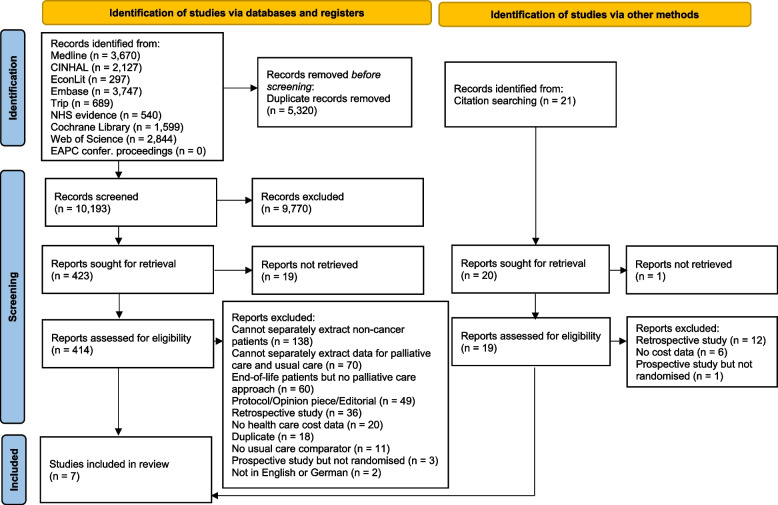
PRISMA flow diagram

The search yielded 15,513 studies. After removing 5,320 duplicates, 10,193 studies were identified for the title and abstract screening. Reference list checking yielded 21 studies. 433 full-text manuscripts were screened, and 7 articles met the inclusion criteria while 426 articles were excluded. Fig. 1 provides details on the study selection process.

### Study characteristics

Table 2 provides details for the studies included in the review. The included studies examined a range of disease groups. Two studies examined heart failure [20, 21], three studies examined multiple sclerosis and other neurological conditions [22–24], one study examined AIDS [25] and one a mixture of diseases in older people [26]. All the studies evaluated specific palliative care interventions. Nearly all of these specific palliative care interventions were delivered at patients’ homes. The only exception was an intervention that combined early palliative care with motivational interviewing delivered during clinics at an HIV care facility [25]. In terms of country, three studies were from the UK [22, 24, 26], one from the USA [25] and one each from Italy [23], Sweden [20], and Hong Kong [21]. The data collection period for most studies was 2010 to 2020 with only one study using data from 2000 to 2010 [22]. There was a total of 416 patients in the palliative care approach group and 389 patients in the usual care group.

**Table 2:** Characteristics and results of the included studies

Reference, Country, Study quality	Palliative care approach	Measurement period	Study design	Diagnosis	Number of patients		Indicative results for effect measures ▲ = palliative care improves outcome ▼ = palliative care worsens outcome ▬ = no difference		Results for cost measures ▼ = palliative care is cost-saving ▲ = palliative care is cost-increasing ▬ = no difference	
					with PC	w/o PC				
Higginson 2009 [22], UK, High	Short-term comprehensive integrated person-centred specialist palliative care delivered at home and in hospital	12 weeks	Randomized fast-track Phase II controlled trial from 2005 to 2006	Multiple sclerosis	26	26	▬	Palliative Care Outcome Scale (POS-8)	▼	Total costs: diff. in means = –£1,789 (95% CI = -5,224 to 1,902)
							▲	Zarit Carer Burden Inventory (ZBI)		
Phillips 2022 [25], USA, High	Early palliative care combined with motivational interviewing with focus on disease adjustment, self-care and advanced care planning	12 months	Randomized controlled trial with two arms from 2012 to 2017	AIDS	61	60	▬	Quality-adjusted life years (QALY)	▼	Hospital costs: mean difference = –$16,505 (p-val. = 0.03)
									▼	Emergency Department costs:mean difference = –$3,852(p-val. = 0.047)
									▬	Clinic costs:mean difference = –$31(p-value = 0.87)
									▼	Total costs (excl. pharmacy costs):adjusted mean ratio = 0.67(95% CI = 0.15 to 0.93)
Rosato 2021 [23], Italy, High	Home-based general palliative care not intended to replace existing services	6 months	Randomized single-blind controlled trial in 2015	Multiple sclerosis	50	26	▬	Quality-adjusted life years (QALY)	▬	Total costs: adj. mean cost difference = –EUR394 (95% CI = -EUR3,629 to EUR2,461)
							▲	Palliative Care Outcome Scale-Symptoms-MS (POS-S-MS)		
Sahlen 2016 [20], Sweden, High	Integrated palliative and heart failure care using structured person-centred care at home with easy access to care	6 months	Randomized controlled trial with two arms from 2011 to 2013	Heart failure	36	36	▲	Quality-adjusted life years (QALY)	▬	Total costs: diff. in means = –EUR1,649 (not significant)
Evans 2021[26], UK, Medium	Short-term comprehensive, integrated person-centred specialist palliative and supportive care delivered at home	12 weeks	Randomized single-blind controlled trial from 2014 to 2016	Mixed (Circulat., respirat., endocrine, neurologic. diseases; dementia)	24	26	▬	Quality-adjusted life years (QALY)	▬	Total costs: diff. in means = –£28 (95% CI = -£3,587 to £3,531)
							▲	Integrated Palliative Care Outcome Scale (IPOS)		
							▬	Zarit Carer Burden Inventory (ZBI)		
Gao 2020 [24], UK, Medium	Short-term comprehensive, integrated person-centred specialist palliative care delivered at home	12 weeks	Randomized Phase III clinical trial from 2015 to 2017	Multiple sclerosis, Parkinson,other neurologic	176	174	▬	Quality-adjusted life years (QALY)	▬	Total costs: difference in changes = -£562 (p-value = 0.12)
							▬	Integrated Palliative Care Outcome Scale (IPOS)		
							▬	Zarit Carer Burden Inventory (ZBI)		
Wong 2018 [21], Hong Kong, Medium	Transitional home-based palliative end-stage heart failure programme	12 weeks	Randomized controlled trial with two arms from 2013 to 2015	Heart failure	43	41	▬	Quality-adjusted life years (QALY)	▼	Total costs: diff. in means = –HK$26,084

*PC* Palliative care, *CI* Confidence interval, *SD* Standard deviation

### Study quality

Table 3 presents the results of the quality assessment. Four studies were assessed as high quality and three as medium quality. Studies with more than one issue on the 10-point Drummond checklist (Reference) where judged as medium quality.

**Table 3:**
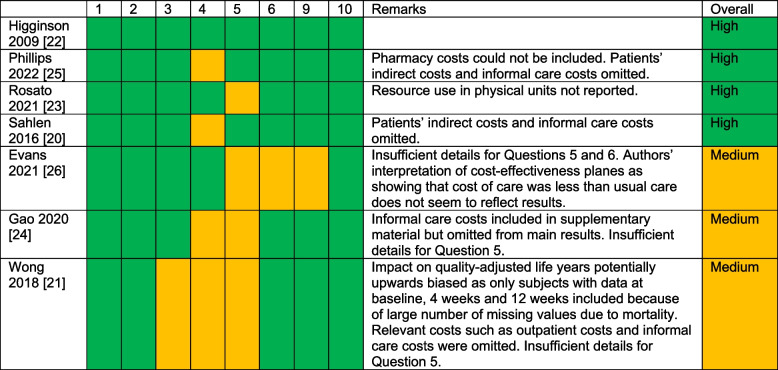
Quality assessment of the included studies using relevant questions on the Drummond checklist

Question 1: Was a well-defined question posed in answerable form? Question 2: Was a comprehensive description of the competing alternatives given? Question 3: Was the effectiveness of the programmes or services established? Question 4: Were all the important and relevant costs and consequences for each alternative identified? Question 5: Were costs and consequences measured accurately in appropriate physical units before valuation? Question 6: Were costs and consequences valued credibly? Question 9: Was uncertainty in the estimates of costs and consequences adequately characterized? Question 10: Did the presentation and discussion of study results include all issues of concern to users?

### Results of syntheses

Overall, we found that a palliative care approach is cost saving or cost neutral. Table 2 presents detailed results for the individual studies, including graphical indicators showing whether a result for a certain outcome suggests that a palliative care approach is cost saving, cost neutral or cost increasing. In terms of outcomes, five out of the 10 outcomes show cost savings, and the other five outcomes show cost neutrality. Aggregating findings at study level, three of the seven studies suggest that a palliative care approach is cost saving [21, 22, 25] while four suggest it is cost neutral. There is no clear pattern in terms of disease group: one study examining heart failure finds cost savings [21] while the other one finds cost neutrality [20]; one study examining multiple sclerosis finds cost savings [22] while the other two find cost neutrality [23, 24]. All three multiple sclerosis studies had a relatively short measurement period of 12 weeks [22–24] but the two heart failure studies had different measurement periods, with cost savings found for a relatively short measurement period of 12 weeks [21] and cost neutrality found for a longer measurement period of 6 months [20]. On the other hand, the study with the longest measurement period of 12 months finds cost savings [25].

To check whether cost savings are achieved at the expense of care quality, we examine the effect measures reported in the studies. Table 2 lists these measures and provides graphical indicators showing whether a result for a certain effect measure suggests that a palliative care approach improves patient outcomes, makes no difference or worsens patient outcomes. Across all effect measures, four out of 13 outcomes suggest that a palliative care approach improves patient outcomes, and the remaining nine outcomes suggest that it makes no difference.

**Fig. 2:**
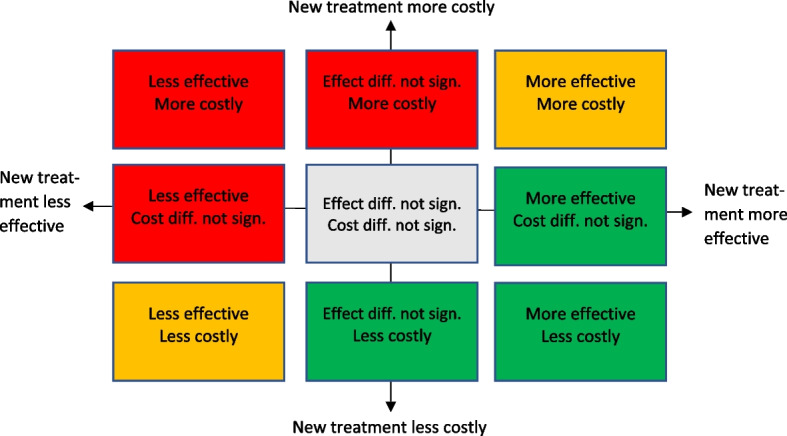
Generic cost-effectiveness plane

To graphically present the relationships between the cost results and the patient outcome results within and between the studies, we use a tool commonly used in economic evaluation: the cost-effectiveness plane. Fig. 2 presents a generic cost-effectiveness plane. On the horizontal axis it shows the difference in effect, i.e. the difference in a specific patient outcome measure, between the new treatment and the usual treatment and on the vertical axis the difference in cost between the new treatment and the usual treatment. The difference in effect between the new treatment and the usual treatment could be not statistically significant, statistically significantly negative or statistically significantly positive and the same for the difference in cost between the new treatment and the usual treatment. Thus, there are nine possible combinations of cost and effect differences which are depicted using colour coding.

The green boxes indicate desirable combinations of cost and effect differences: the new treatment is both more effective and less costly or the new treatment is more effective with no statistically significant cost difference or the new treatment is less costly with no statistically significant effect difference. The red boxes indicate combinations that would lead to a rejection of the new treatment: the new treatment is both less effective and more costly or the new treatment is less effective with no statistically significant cost difference or the new treatment is more costly with no statistically significant effect difference. The yellow boxes indicate combinations that require difficult decisions to be made: the new treatment is both more effective and more costly or the new treatment is both less effective and less costly. Many new medical technologies fall into the former category, so a decision maker needs to decide whether the improved effectiveness is worth the additional costs. The grey box indicates that neither the effect difference nor the cost difference is statistically significant.

**Fig. 3:**
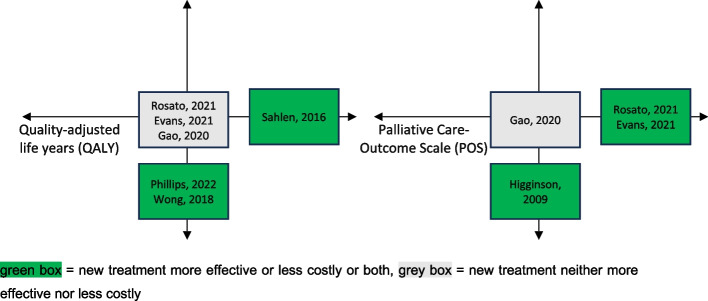
Location of studies on the cost-effectiveness plane: patient outcomes

**Fig. 4:**
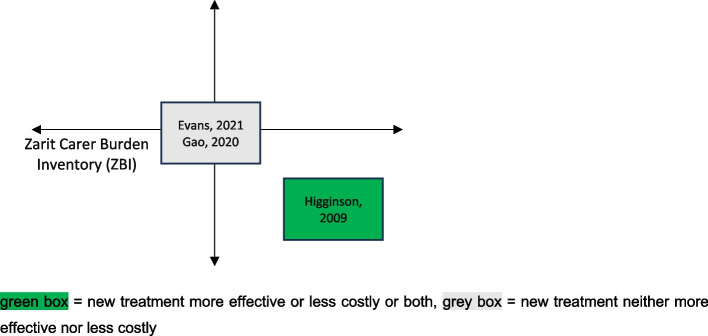
Location of studies on the cost-effectiveness plane: carer outcomes

Figures 3 and 4 place the included studies on the cost-effectiveness plane. Fig. 3 shows the position of each study with respect to the patient outcomes reported while Fig. 4 shows the position for the carer outcomes reported. The patient outcomes are quality-adjusted life years (QALY) on the left-hand side of Fig. 3 and different versions of the Palliative Care Outcome Scale (POS) on the right-hand side of Fig. 3. The carer outcome in Fig. 4 is the Zarit Carer Burden Inventory (ZBI). Note that not all studies report all outcomes, so different studies are presented on each of the three cost-effectiveness planes.

The results are spread across four of the nine possible combinations: a palliative care approach (i) improves carer outcomes and is cost saving [22] (ii) improves patient outcomes while being cost-neutral [20, 23, 26], (ii) is cost-saving while making no difference to patient outcomes [21, 22, 25] and (iii) is cost-neutral and makes no difference to patient or carer outcomes [23, 24, 26]. Thus, there is no suggestion that cost savings are accompanied by worse outcomes for patients or carers. The ideal situation of achieving both cost savings and improved outcomes for patients and carers, however, is rare.

## Discussion

In a non-cancer population, this is the first systematic review of RCTs that has demonstrated a palliative care approach is cost-saving or at least cost-neutral. Cost savings are achieved without worsening outcomes for patients and carers.

These findings complement the findings in the only other systematic review that has focused on non-cancer patients with life-limiting illnesses. Quinn et al. [10] found that palliative care was associated with less health care use [10]. However, their measures of health care use were limited to emergency department use and hospitalization, which we would expect to be lower as palliative care is often home-based. Our findings show that with a less partial measure – total health care costs – the impact of palliative care is less clear.

Two recent reviews that included both cancer and non-cancer patients report findings for both health care use and total health care costs and found moderate evidence for reductions in hospital use but weaker evidence for palliative care reducing total health care costs [6, 27]. Kavalieratos et al. [27] stress that none of the RCTs in the review found that palliative care increases total health care costs [27] while both reviews found weak evidence of palliative care improving outcomes for patients and carers [6, 27]. Thus, despite the difference in the patient population – in both reviews around 70% of RCTs included in the review included patients with cancer – their findings are in line with our findings that a palliative care approach is at best cost-saving and at worst cost-neutral while at best improving and at worst not affecting patient and carer outcomes. The two Cochrane reviews found that the evidence on total health care costs is inconclusive but in line with our results they found that palliative care is at worst cost-neutral [4, 5].

Our findings differ from the findings of reviews that include observational studies in addition to RCTs for both cancer and non-cancer patients in that they tend to report that a palliative care approach reduces total health care costs [8, 9, 28, 29]. The majority of the evidence in these reviews comes from observational studies that at best control for confounding using propensity score methods [9], multivariate regression analysis [29] or before-and-after comparisons [8, 29]. Observational methods can overestimate beneficial treatment effects compared to RCTs [30], which might explain our more nuanced findings for RCTs.

The evidence included in the review had a few limitations. Firstly, except for two studies [22, 26] all the studies adopted a perspective that was limited to the health and social care sector or even to only the health sector. Such a narrow perspective excludes, for example, informal care costs. It is not implausible that a palliative care approach results in a higher burden for informal caregivers. This issue of most studies ignoring the cost to patients, families and caregivers was noted in an earlier review that examined both cancer and non-cancer patients and only included RCTs [7] and still observed in a recent methodological review [31].

Secondly, most of the studies included in this review appear to be underpowered to detect cost differences between palliative care and usual care. Trials tend to be designed to detect a difference in the clinical outcomes. The sample size required to detect a difference in costs will be greater due to typically higher variance in the resource use measures [32]. Therefore, some of the outcomes showing cost-neutrality could be due to low power rather than evidence of absence of cost savings.

Thirdly, there is a lack of evidence on the impact of a palliative care approach in patients with dementia, who account for 12.2% of adults in need of palliative care, the fourth largest disease group after cancer, HIV and cerebrovascular disease [1]. Finally, all the studies included in the review were conducted in high-income countries. Healthcare systems in low- and middle-income countries are likely to be different, so our findings might not be generalisable to other parts of the world.

This review has several limitations. Because of time constraints, only 36% of studies were quality assessed by two reviewers. However, assessing only a subset of studies by two reviewers is common practice for systematic reviews. Furthermore, we only included studies in English or German, so we might have excluded relevant studies, especially from low- and middle-income countries.

Our study has implications for practice, policy and future research. Our finding that at worst palliative care is cost-neutral without affecting outcomes of patients and carers but might also be cost-saving at no detriment to patients and carers lends support to calls to increase palliative care provision globally. Currently, palliative care is provided to less than 14% of those that need it globally [1].

At the practice level, our results lend support to clinicians’ efforts to ensure appropriate palliative care provision for their patients as in addition to potentially improving the experience of patients with non-cancer life-limiting illnesses [10, 27], palliative care might reduce health care costs for this patient population.

Future studies need to adopt a wider societal rather than a narrow health and social care perspective as costs incurred by patients, families and caregivers are likely to be substantial.

## Conclusions

A palliative care approach in patients with non-cancer life-limiting illnesses might generate cost savings that are unlikely to be accompanied by worse outcomes for patients and carers. At worst a palliative care approach is cost-neutral but might improve outcomes for patients and carers. These findings support global policy to increase palliative care provision for non-cancer illness. Future research should focus on trials that are powered for an economic evaluation, include a societal perspective and examine a wider range of conditions, especially dementia, across all settings and countries where palliative care is delivered.

### Other information

The protocol for this systematic review was registered with PROSPERO in August 2020 under registration number CRD42020191082 and can be found at https://www.crd.york.ac.uk/prospero/display_record.php?ID=CRD42020191082. The protocol has since been updated by limiting the included studies to only Randomised Controlled Trials.

## Data Availability

The authors are willing to share the data extraction forms at reasonable request. Requests should be submitted to the corresponding author.

## References

[1] Connor S, Bermedo MS, World Palliative Care Alliance. World Health Organization global atlas of palliative care at the end of life. London: Worldwide Palliative Care Alliance; 2020.

[2] Fassbender K, Fainsinger RL, Carson M, Finegan BA. Cost Trajectories at the end of life: the Canadian experience. J Pain Symptom Manage. 2009;38(1):75–80. 19615630 10.1016/j.jpainsymman.2009.04.007

[3] Howdon D, Rice N. Health care expenditures, age, proximity to death and morbidity: implications for an ageing population. J Health Econ. 2018;57:60–74. 29182935 10.1016/j.jhealeco.2017.11.001

[4] Gomes B, Calanzani N, Curiale V, McCrone P, Higginson IJ, de Brito M. Effectiveness and cost-effectiveness of home palliative care services for adults with advanced illness and their caregivers. Cochrane Database Syst Rev. 2013;2013(6):CD007760. 23744578 10.1002/14651858.CD007760.pub2 PMC4473359

[5] Bajwah S, Oluyase AO, Yi D, Gao W, Evans CJ, Grande G, Todd C, Costantini M, Murtagh FE, Higginson IJ. The effectiveness and cost-effectiveness of hospital-based specialist palliative care for adults with advanced illness and their caregivers. Cochrane Database Syst Rev. 2020;9(9):CD012780. 32996586 10.1002/14651858.CD012780.pub2 PMC8428758

[6] Singer AE, Goebel JR, Kim YS, Dy SM, Ahluwalia SC, Clifford M, Dzeng E, O’Hanlon CE, Motala A, Walling AM, et al. Populations and interventions for palliative and end-of-life care: a systematic review. J Palliat Med. 2016;19(9):995–1008. 27533892 10.1089/jpm.2015.0367 PMC5011630

[7] Zimmermann C, Riechelmann R, Krzyzanowska M, Rodin G, Tannock I. Effectiveness of specialized palliative care: a systematic review. JAMA. 2008;299(14):1698–709. 18398082 10.1001/jama.299.14.1698

[8] Gonzalez-Jaramillo V, Fuhrer V, Gonzalez-Jaramillo N, Kopp-Heim D, Eychmüller S, Maessen M. Impact of home-based palliative care on health care costs and hospital use: a systematic review. Palliat Support Care. 2021;19(4):474–87. 33295269 10.1017/S1478951520001315

[9] May P, Normand C, Cassel JB, Del Fabbro E, Fine RL, Menz R, Morrison CA, Penrod JD, Robinson C, Morrison RS. Economics of palliative care for hospitalized adults with serious illness: a meta-analysis. JAMA Intern Med. 2018;178(6):820–9. 29710177 10.1001/jamainternmed.2018.0750 PMC6145747

[10] Quinn KL, Shurrab M, Gitau K, Kavalieratos D, Isenberg SR, Stall NM, Stukel TA, Goldman R, Horn D, Cram P, et al. Association of receipt of palliative care interventions with health care use, quality of life, and symptom burden among adults with chronic noncancer illness: a systematic review and meta-analysis. JAMA. 2020;324(14):1439–50. 33048152 10.1001/jama.2020.14205 PMC8094426

[11] Bach PB, Schrag D, Begg CB. Resurrecting treatment histories of dead patients: a study design that should be laid to rest. JAMA. 2004;292(22):2765–70. 15585737 10.1001/jama.292.22.2765

[12] Popay J, Roberts H, Sowden A, Petticrew M, Arai L, Rodgers M, Britten N, Roen K, Duffy S. Guidance on the conduct of narrative synthesis in systematic reviews. Prod ESRC Methods Program Version. 2006;1(1):b92.

[13] Page MJ, McKenzie JE, Bossuyt PM, Boutron I, Hoffmann TC, Mulrow CD, Shamseer L, Tetzlaff JM, Akl EA, Brennan SE, et al. The PRISMA 2020 statement: an updated guideline for reporting systematic reviews. BMJ. 2021;372:n71. 33782057 10.1136/bmj.n71 PMC8005924

[14] Connor SR, Bermedo M: Global atlas of palliative care at the end of life: Worldwide Palliative Care Alliance. London: World Health Organization; 2014.

[15] Drummond MF, Sculpher MJ, Claxton K, Stoddart GL, Torrance GW. Methods for the economic evaluation of health care programmes. Oxford: Oxford University Press; 2015.

[16] Harris I, Murray SA. Can palliative care reduce futile treatment? A systematic review. BMJ Support Palliat Care. 2013;3(4):389–98. 24950518 10.1136/bmjspcare-2012-000343

[17] Janssens JP, Weber C, Herrmann FR, Cantero C, Pessina A, Matis C, Merlet Viollet R, Boiche-Brouillard L, Stirnemann J, Pautex S. Can early introduction of palliative care limit intensive care, emergency and hospital admissions in patients with severe chronic obstructive pulmonary disease? A pilot randomized study. Respiration. 2019;97(5):406–15. 30650418 10.1159/000495312

[18] Pattenden JF, Mason AR, Lewin RJP. Collaborative palliative care for advanced heart failure: outcomes and costs from the ‘Better Together’ pilot study. BMJ Support Palliat Care. 2013;3(1):69–76. 24644330 10.1136/bmjspcare-2012-000251

[19] Slavin RE. Best evidence synthesis: an intelligent alternative to meta-analysis. J Clin Epidemiol. 1995;48(1):9–18. 7853053 10.1016/0895-4356(94)00097-A

[20] Sahlen K-G, Boman K, Brännström M. A cost-effectiveness study of person-centered integrated heart failure and palliative home care: Based on a randomized controlled trial. Palliat Med. 2016;30(3):296–302. 26603186 10.1177/0269216315618544

[21] Wong FKY, So C, Ng AYM, Lam P-T, Ng JSC, Ng NHY, Chau J, Sham MMK. Cost-effectiveness of a transitional home-based palliative care program for patients with end-stage heart failure. Palliat Med. 2018;32(2):476–84. 28434275 10.1177/0269216317706450

[22] Higginson IJ, McCrone P, Hart SR, Burman R, Silber E, Edmonds PM. Is short-term palliative care cost-effective in multiple sclerosis? A randomized phase II trial. J Pain Symptom Manage. 2009;38(6):816–26. 19833477 10.1016/j.jpainsymman.2009.07.002

[23] Rosato R, Pagano E, Giordano A, Farinotti M, Ponzio M, Veronese S, Confalonieri P, Grasso MG, Patti F, Solari A. Living with severe multiple sclerosis: cost-effectiveness of a palliative care intervention and cost of illness study. Mult Scler Relat Disord. 2021;49:102756. 33486403 10.1016/j.msard.2021.102756

[24] Gao W, Wilson R, Hepgul N, Yi D, Evans C, Bajwah S, Crosby V, Wilcock A, Lindsay F, Byrne A, et al. Effect of short-term integrated palliative care on patient-reported outcomes among patients severely affected with long-term neurological conditions: a randomized clinical trial. JAMA Netw Open. 2020;3(8):e2015061–e2015061. 32857151 10.1001/jamanetworkopen.2020.15061 PMC7455856

[25] Phillips V, Quest TE, Higgins M, Marconi VC, Balthazar MS, Holstad M. A cost-effective analysis of motivational interviewing with palliative care versus usual care: results from the living well project. AIDS Behav. 2023;27(4):1259–68. 36334215 10.1007/s10461-022-03862-8 PMC10832615

[26] Evans CJ, Bone AE, Yi D, Gao W, Morgan M, Taherzadeh S, Maddocks M, Wright J, Lindsay F, Bruni C, et al. Community-based short-term integrated palliative and supportive care reduces symptom distress for older people with chronic noncancer conditions compared with usual care: a randomised controlled single-blind mixed method trial. Int J Nurs Stud. 2021;120:103978. 34146843 10.1016/j.ijnurstu.2021.103978

[27] Kavalieratos D, Corbelli J, Zhang D, Dionne-Odom JN, Ernecoff NC, Hanmer J, Hoydich ZP, Ikejiani DZ, Klein-Fedyshin M, Zimmermann C, et al. Association between palliative care and patient and caregiver outcomes: a systematic review and meta-analysis. JAMA. 2016;316(20):2104–14. 27893131 10.1001/jama.2016.16840 PMC5226373

[28] Luta X, Ottino B, Hall P, Bowden J, Wee B, Droney J, Riley J, Marti J. Evidence on the economic value of end-of-life and palliative care interventions: a narrative review of reviews. BMC Palliat Care. 2021;20:89. 34162377 10.1186/s12904-021-00782-7 PMC8223342

[29] Smith S, Brick A, O’Hara S, Normand C. Evidence on the cost and cost-effectiveness of palliative care: a literature review. Palliat Med. 2014;28(2):130–50. 23838378 10.1177/0269216313493466

[30] Dahabreh IJ, Sheldrick RC, Paulus JK, Chung M, Varvarigou V, Jafri H, Rassen JA, Trikalinos TA, Kitsios GD. Do observational studies using propensity score methods agree with randomized trials? A systematic comparison of studies on acute coronary syndromes. Eur Heart J. 2012;33(15):1893–901. 22711757 10.1093/eurheartj/ehs114 PMC3409422

[31] Parackal A, Ramamoorthi K, Tarride J-E. Economic evaluation of palliative care interventions: a review of the evolution of methods from 2011 to 2019. Am J Hosp Palliat Med. 2022;39(1):108–22. 10.1177/10499091211011138 34024147

[32] Briggs A. Economic evaluation and clinical trials: size matters. BMJ : British Medical Journal. 2000;321(7273):1362. 11099268 10.1136/bmj.321.7273.1362 PMC1119102

